# Epithelial immunotherapy for food allergy in children: a systematic review and meta-analysis

**DOI:** 10.3389/fimmu.2024.1510653

**Published:** 2024-12-23

**Authors:** Bin Chen, Hu Gao, Xihong Li, Zhuan Zou, Shanshan Wu, Fajuan Tang

**Affiliations:** ^1^ Department of Pediatrics, West China Second University Hospital, Sichuan University, Chengdu, China; ^2^ Key Laboratory of Birth Defects and Related Diseases of Women and Children, Sichuan University, Chengdu, China

**Keywords:** epicutaneous immunotherapy, food allergy, desensitization, adverse reaction, children

## Abstract

**Objectives:**

Traditional methods of treating allergies primarily revolve around avoiding allergens and promptly using rescue medications when allergic symptoms occur. However, this approach is known for its inefficiency and limited success in achieving long-term relief. Our aim was to conduct a comprehensive analysis of previously published randomized controlled trials (RCTs) that explore the effectiveness and safety of epicutaneous immunotherapy (EPIT) as a means to manage food allergies in children.

**Methods:**

We conducted a comprehensive search across multiple databases, including PubMed, Web of Science, Embase, and Cochrane Library, to identify RCTs comparing EPIT versus placebo for the management of allergen-triggered allergic reactions in children. Only RCTs published in English that evaluated the efficacy and safety of EPIT in pediatric patients with allergic diseases were considered eligible for inclusion. The quality assessment of the included studies was performed using the Cochrane risk-of-bias tool. The analysis comprised of seven RCTs involving a total of 1141 participants. The meta-analysis demonstrated that EPIT significantly facilitated desensitization in patients with food allergy (RR: 2.12, 95% CI: 1.74-2.59, P = 0.296, I² = 17.5%), particularly in individuals with peanut allergy (RR: 2.29, 95% CI: 1.83-2.86, P = 0.463, I² = 0%). However, it is important to note that EPIT was associated with an increased occurrence of treatment-related adverse events (TRAEs; RR: 1.24, 95% CI: 1.14-1.34, P < 0.01, I² = 99.2%). Notably, there were no notable disparities in the frequency of serious adverse events or utilization of rescue medications between the EPIT and placebo groups. EPIT may potentially induce desensitization of peanut allergy in children, but also carries an elevated risk of TRAEs.

## 1 Introduction

Food allergy is a significant concern for public health and can potentially be life-threatening, affecting approximately 5% of adults and up to 8% of children in Western countries (1, 2). The prevalence rate of cow’s milk allergy is estimated to be around 2% among children aged five years or younger in the United States (3), making it a crucial issue contributing to fatal allergic reactions among young children in both the United States (4, 5) and European nations (6). Peanut allergy also represents the most prevalent life-threatening food allergy, affecting more than 2% of children in the United States (7). Milk and peanuts are crucial components of a balanced diet, and successfully avoiding these allergens can be extremely difficult. Even the slightest exposure carries the risk of accidental allergies, leading to frequent visits to the emergency room. Severe cases can even be life-threatening (4, 8), resulting in illness and significantly impacting an individual’s quality of life (9). The current standard approach for managing food allergies involves strict avoidance of allergens (10) and promptly using rescue medications when allergic symptoms occur.

The immunotherapy for food allergies involves modifying the immune response through repeated exposure to increasing amounts of allergenic foods (11). The goal is to reduce the risk associated with accidental ingestion by raising the threshold at which a patient experiences an allergic reaction (12). Presently employed immunotherapy methods, including oral immunotherapy (OIT), sublingual immunotherapy (SLIT), and epicutaneous immunotherapy (EPIT), aim to induce immune tolerance towards the allergen by administering a specific dose repeatedly. However, each technique utilizes a distinct administration route and exhibits varying levels of effectiveness and safety. The efficacy of OIT in enhancing the tolerance level of individuals with persistent food allergies has been demonstrated through the administration of higher doses of sensitized food orally (13). This innovative approach has received FDA breakthrough-therapy designation (14); however, its widespread adoption is impeded by the occurrence of severe systemic reactions (15). Notably, gastrointestinal symptoms (such as nausea, vomiting, stomach discomfort, and acid reflux) are the primary reasons for discontinuing OIT. In severe cases, immediate measures such as administering epinephrine may be necessary (16), leading to its near abandonment. SLIT relies on the utilization of Langerhans cells (LCs), which are immune cells that foster tolerance and can be found in the oral mucosa (17). However, due to limitations in available extracts and the ability for fluid absorption beneath the tongue, SLIT is administered at a dosage that is 1000 times lower than OIT (18). Although SLIT treatment demonstrates improved safety, it shows diminished efficacy in desensitizing individuals to peanut and milk allergens compared to OIT (19, 20).

EPIT is a non-invasive technique that delivers small amounts of allergens to the outermost layer of the skin using a patch. This approach specifically targets regions of the skin with a high density of antigen-presenting cells (APCs) (21, 22). Subsequently, these APCs migrate to adjacent lymph nodes where they initiate an immune response and stimulate production of regulatory cytokines, ultimately leading to modified reactions from allergen-specific T-cells (23, 24). The delivery of allergens through EPIT is restricted to active immune cells located in the epidermis, thereby minimizing the likelihood of significant allergen release into the bloodstream due to the absence of blood vessels within this layer (25). Rendering it an attractive method for augmenting efficacy and shortening treatment duration. Through repeated exposures facilitated by this skin patch, the immune system is modulated, resulting in a reduction of symptoms and severity of food allergies, an increase in individual tolerance, and a decrease in sensitivity over time (26, 27). Xiong et al. conducted a systematic review, which revealed that EPIT may induce desensitization in peanut allergy and increase the likelihood of treatment-related adverse events (28). This finding is consistent with the systematic review and meta-analysis performed by Banatwala et al. (29) However, none of these studies specifically focused on pediatric populations. It is imperative to conduct an up-to-date systematic review of EPIT in children.

This systematic review and meta-analysis aim to comprehensively synthesize published randomized controlled trials (RCTs) investigating the efficacy and safety of EPIT as a therapeutic intervention for pediatric food allergy.

## 2 Methods

### 2.1 Protocol and registration

This investigation strictly adhered to the guidelines outlined in the Preferred Reporting Items for Systematic Reviews and Meta-Analyses (PRISMA) (30). Furthermore, the research plan was officially registered with the International Prospective Register of Systematic Reviews (PROSPERO; registration number CRD 42024538489) on February 5, 2024.

### 2.2 Search strategy

We conducted a comprehensive search across renowned databases, including PubMed, EMBASE, Cochrane Library, and Web of Science, to identify English-language studies published from the inception until May 5, 2024. Our search terms included “epicutaneous immunotherapy,” “immunotherapy,” “EPIT,” “peanut allergy,” “milk allergy,” “nut allergy,” “egg allergy,” “food allergy”, “children” “young” and “kids”. The search strategy was developed based on the MeSH Database journals of PubMed and subsequently applied to other databases. Emphasizing human studies exclusively, we limited our inclusion criteria to those written solely in English. Furthermore, we meticulously scrutinized the reference lists of all primary studies available to ensure no pertinent citations were overlooked.

### 2.3 Eligibility criteria

We have developed a “PICOS” (Patient, Intervention, Comparison, Outcome, and Study design) strategy to determine eligibility. The specific criteria are as follows: (1) Participants: Individuals below the age of 18, who have been diagnosed with food allergies or present compelling clinical evidence of food allergies such as peanut, cow’s milk, nuts, and other allergens based on their clinical history and laboratory tests; (2) Treatment: EPIT administered to the intervention group; (3) Control: placebo or allergen avoidance; (4) Outcome: evaluation of food allergy desensitization efficacy and intervention safety; and (5) Study design: limited to RCTs. Exclusion criteria include non-randomized controlled trials, systematic reviews, meta-analyses, narrative reviews, editorials, abstract reports, and case series.

### 2.4 Data collection

The titles, abstracts, and full-texts were meticulously reviewed by two reviewers. Data extraction and risk assessment were independently conducted by the two reviewers. In case of any discrepancies, a third reviewer was consulted for resolution through discussion. A customized data collection form was utilized for the extraction process. If the necessary information was not provided in the original article, efforts were made to contact the authors for its acquisition.

### 2.5 Quality assessment

To evaluate the certainty of evidence utilized in this systematic review and meta-analysis, two reviewers employed a tool provided by the Cochrane Collaboration (31) to examine potential biases present in the included RCTs. The assessment domains encompassed various aspects such as “randomization procedure,” “deviations from intended interventions,” “unavailable outcome data,” “outcome measurement,” “selection of reported outcomes,” and “overall bias.” Each domain was categorized as either “high risk,” “some concerns,” or “low risk.” Discrepancies were resolved through discussion or consultation with the corresponding author.

### 2.6 Data synthesis and analysis

To analyze continuous outcomes, we aggregated the data by calculating the mean difference or standardized mean difference (SMD) if measurements were on different scales. For dichotomous outcomes, we employed the risk ratio (RR). The selection of a random-effects model or fixed-effects model was contingent upon the observed level of variation among studies. We computed a 95% confidence interval (CI) for each estimate of effect size and determined statistical significance based on a P-value below 0.05. To evaluate heterogeneity in the meta-analysis, we utilized the I^2^ statistic and considered it as low if I^2^ < 50% (32). If sufficient data were available, subgroup analyses would be conducted to examine primary outcomes based on factors such as allergen types and intervention dosages. Sensitivity analyses would be performed to assess the robustness of our findings by excluding studies with a “high” or “unclear” risk of bias in terms of selection, performance, detection, and attrition. Begg’s test and Egger’s test would be utilized to evaluate potential publication bias in primary outcomes. Meta-analyses would be carried out using the STATA software (Stata-Corp LLC, CollegeStation, TX, United States). Additionally, the quality of evidence would be assessed using the GRADE approach which categorizes certainty into high, moderate, low, and very low levels.

## 3 Results

### 3.1 Search results and study characteristics

**Figure 1:**
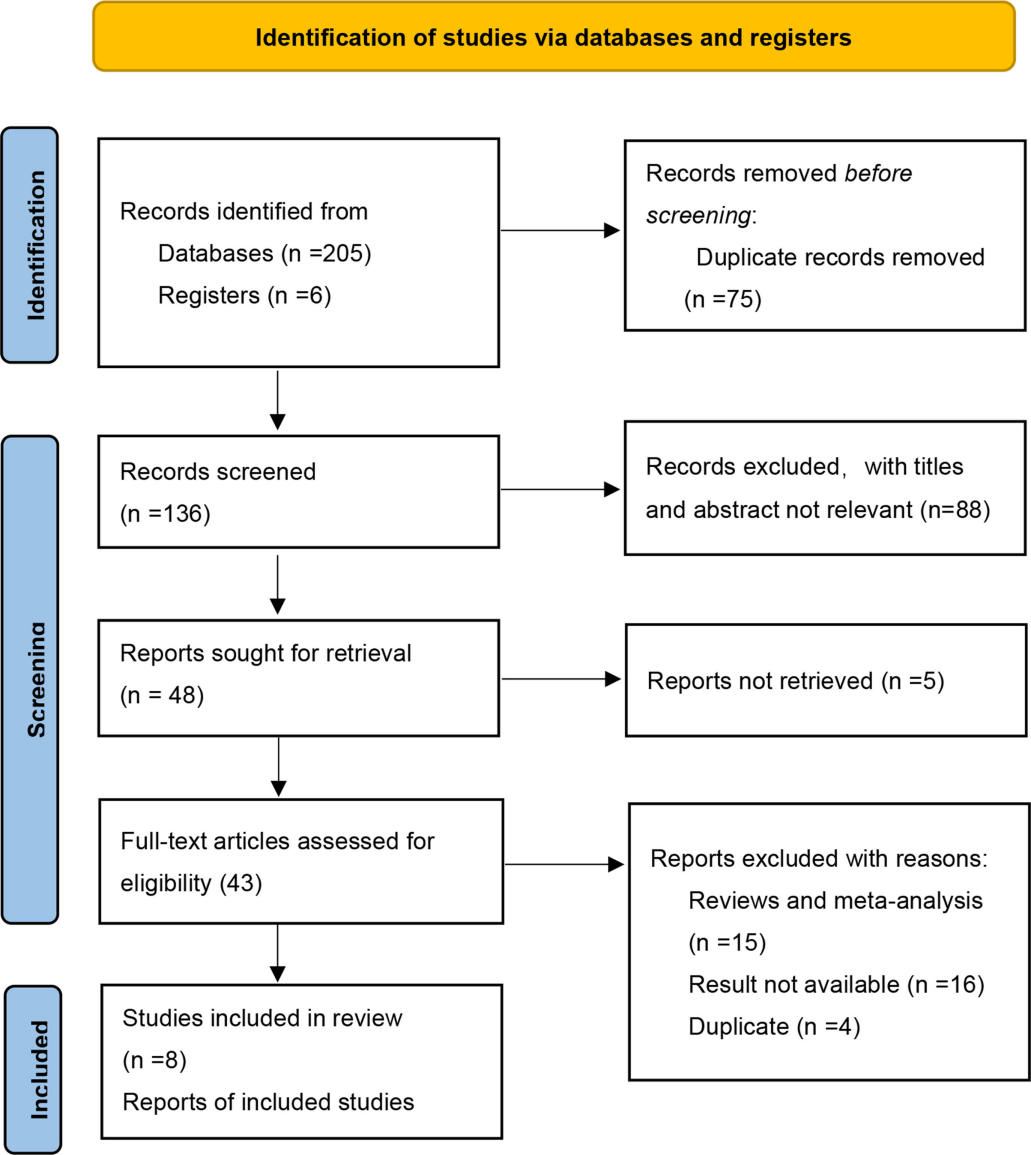
Flow chart of the literature search.

The initial search identified a total of 211 potentially relevant records. Subsequently, a comprehensive full-text screening was conducted on forty-three records, resulting in the inclusion of seven RCTs (33–39) for the final quantitative and qualitative analysis. **Figure 1** presents a flow diagram illustrating the process of study selection.

The characteristics of the studies included in our analysis are summarized in **Table 1**. A total of 1141 participants were enrolled, with 783 assigned to the EPIT group and 358 to the placebo group. Simultaneous multicenter trials were conducted across all continents, including two trials in the USA, one trial in France, and another trial in North America. Most trials (5 out of 7) had an intervention duration of 52 weeks, while the remaining two trials lasted for 9 months and 12 weeks respectively. Three studies focused on cow’s milk allergy and utilized EPIT doses ranging from 150 µg to 1 mg, whereas the other four studies concentrated on peanut allergy with EPIT doses ranging from 50 to 250 µg.

**Table 1:** Baseline characteristics and primary results of included trials.

Author, years	Country	Sample size	Age (years)	Diagnose	Baseline provocation test	Treatment duration	Intervention	Control	Outcomes
Dupont et al. (33)	France	18	3moths-15years	Clinical diagnosis	Positive OFC	12 weeks	Viaskin milk patch (1 mg)	Placebo	DE; LAR; SAE
Sampson et al. (38)	EU, NA	113	6-11	Clinical diagnosis	Positive OFC	12 months	Viaskin peanut patch (50, 100, and 250μg)	Placebo	DE; SAE
Jones et al. (36)	USA	74	4-20	Clinical diagnosis	Positive OFC	52 weeks	Viaskin peanut patch (100 and 250μg)	Placebo	DE; TRAE; LAR; SAR; SAE
Fleischer et al. (34)	Australia, EU, NA	356	4-11	Physician diagnosis	Positive OFC	12 months	Viaskin peanut patch (250μg)	Placebo	DE; TRAE; LAR; SAR; SAE; ES
Spergel et al., (39)	USA	20	4-17	Physician diagnosis	Positive OFC	9 months	Viaskin milk protein patch(500μg)	Placebo	DE; LAR; SAR; SAE
Greenhawt et al. (35)	Australia, EU, NA	362	1-3	Physician diagnosis	Positive OFC	12 months	Viaskin peanut patch (250μg)	Placebo	DE; TRAE; LAR; SAR; SAE; ES
Petroni et al. (37)	NA	198	2-17	Clinical diagnosis	Positive OFC and skin-prick test	12 months	Viaskin milk patch (150, 300, and 500ug)	Placebo	DE; TRAE; LAR; SAR; SAE

EU, Europe; NA, North America; USA, America; OFC, oral food challenge; DE, desensitization; TRAE, treat related adverse event; LAR, Local adverse reactions; SAR, Systemic adverse reactions; SAE, serious adverse events; ES, epinephrine use.

**Figure 2:**
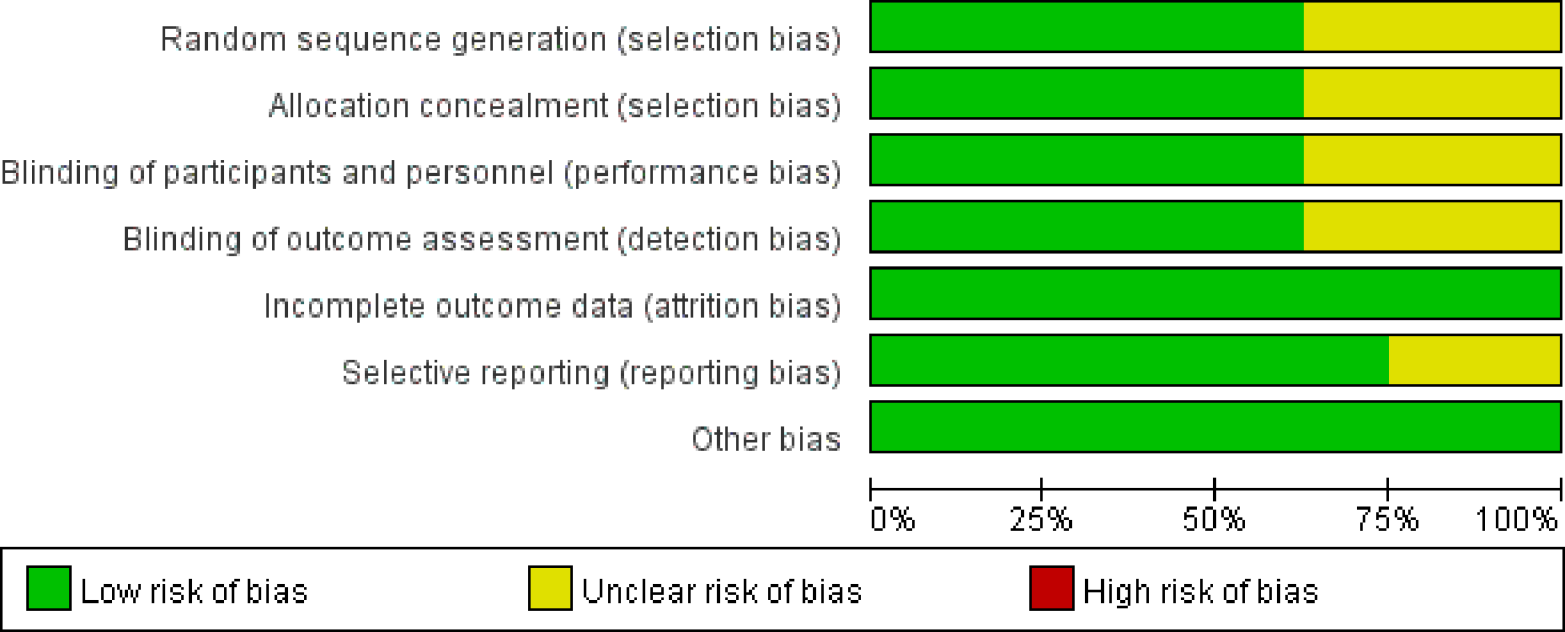
Risk of bias graph.

The risk of bias in the included studies is depicted in **Figures 2**. Following the guidelines outlined in the Cochrane Handbook, four studies demonstrated a low risk of bias (34, 35, 37, 39). Concerns regarding incomplete data on random sequence generation, allocation concealment, blinding of participants and personnel, as well as blinding of outcome assessment were raised for two studies (33, 36). Furthermore, one study was flagged for potential issues related to selective reporting (38).

### 3.2 Primary outcome

#### 3.2.1 Desensitization

**Figure 3:**
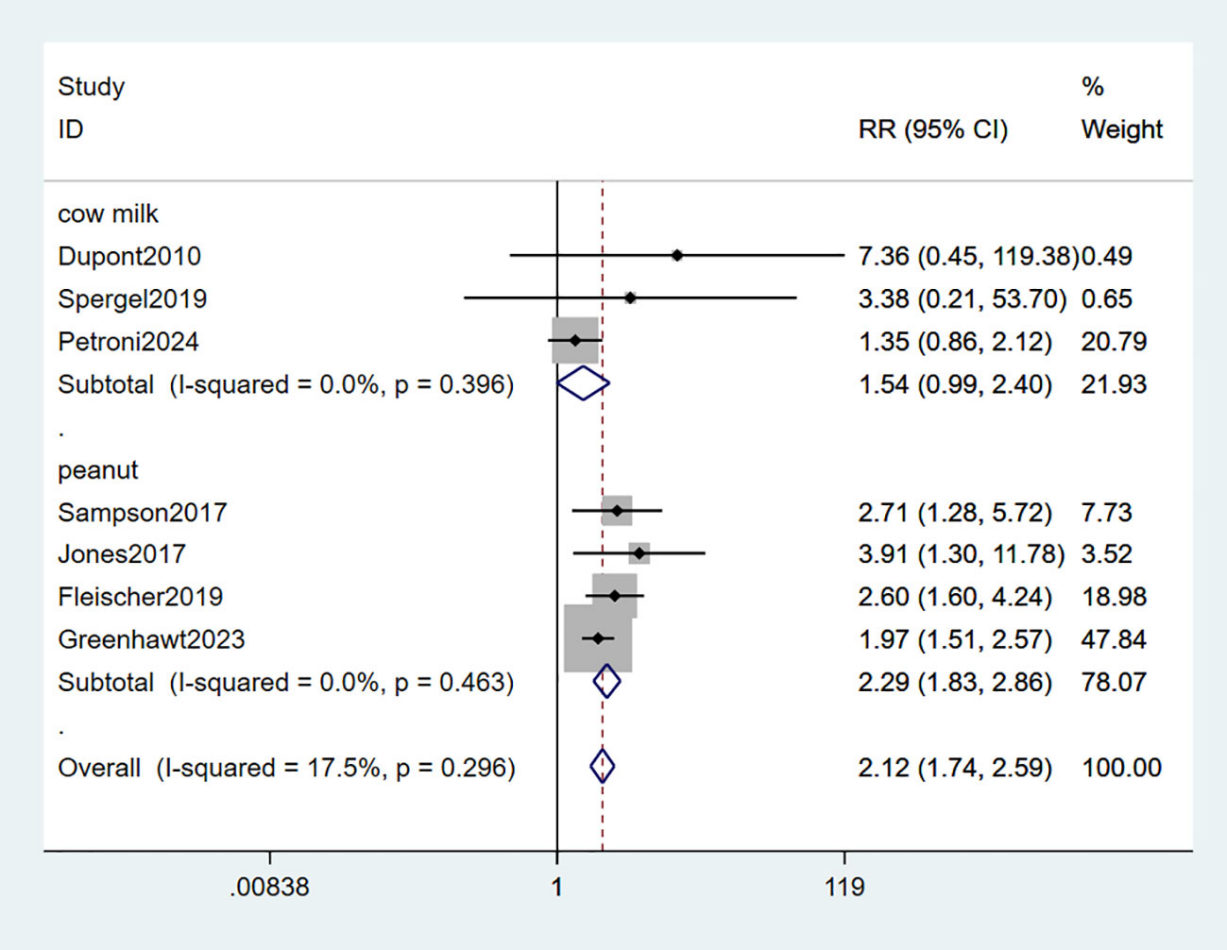
Forest plot of desensitization.

All seven studies included in the analysis provided data on desensitization. Among these, four studies demonstrated an increased ability to tolerate peanut allergies (34–36, 38), while two studies reported an improved tolerance towards milk allergies (33, 37). Only one study specifically assessed the efficacy of EPIT in reducing eosinophil counts to less than 15/hpf from biopsy for milk-induced eosinophilic esophagitis (EOE) (39). The combined data showed a statistically significant tolerance of food allergy in the EPIT group compared with placebo (RR: 2.12, 95% CI: 1.74-2.59, P = 0.296, I² = 17.5%) with minimal heterogeneity observed. Interestingly, all four studies involving peanut patches demonstrated favorable outcomes in terms of desensitization (RR: 2.29, 95% CI: 1.83-2.86, P = 0.463, I² = 0%); however, no study involving milk patches reported any significant improvement in this regard (RR: 1.54, 95% CI: 0.99-2.40, P = 0.396, I² = 0%) (high certainty evidence; **Figure 3**).

#### 3.2.2 Treatment-related adverse events

**Figure 4:**
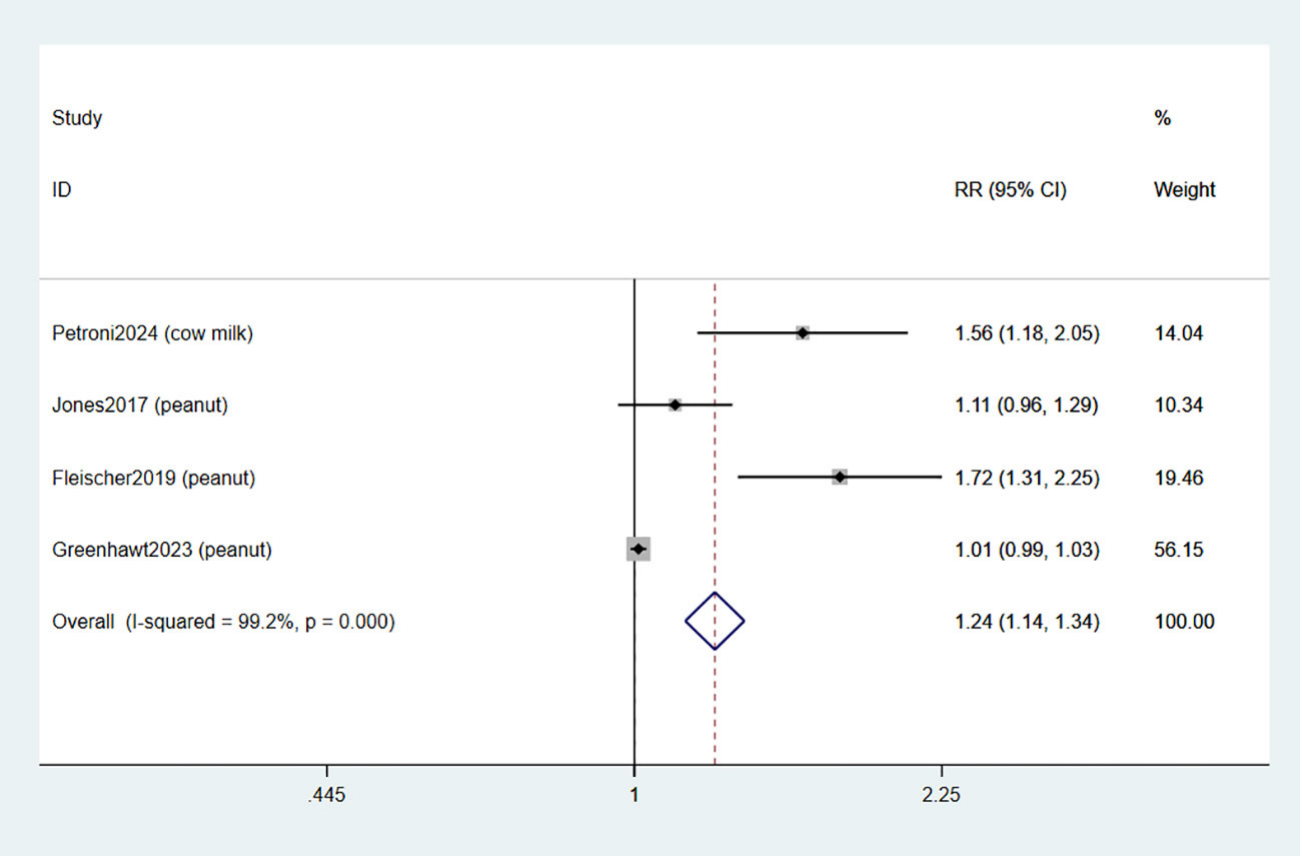
Forest plot of TRAEs.

Four studies documented the occurrence of adverse events related to treatment (TRAE) (34–37). The combined data indicated that EPIT was associated with an increased risk of any TRAE compared to placebo (RR: 1.24, 95% CI: 1.14-1.34, P < 0.01, I^2^ = 99.2%) with a significant level of heterogeneity when compared to placebo (low certainty evidence; **Figure 4**).

#### 3.2.3 Local adverse reactions

**Figure 5:**
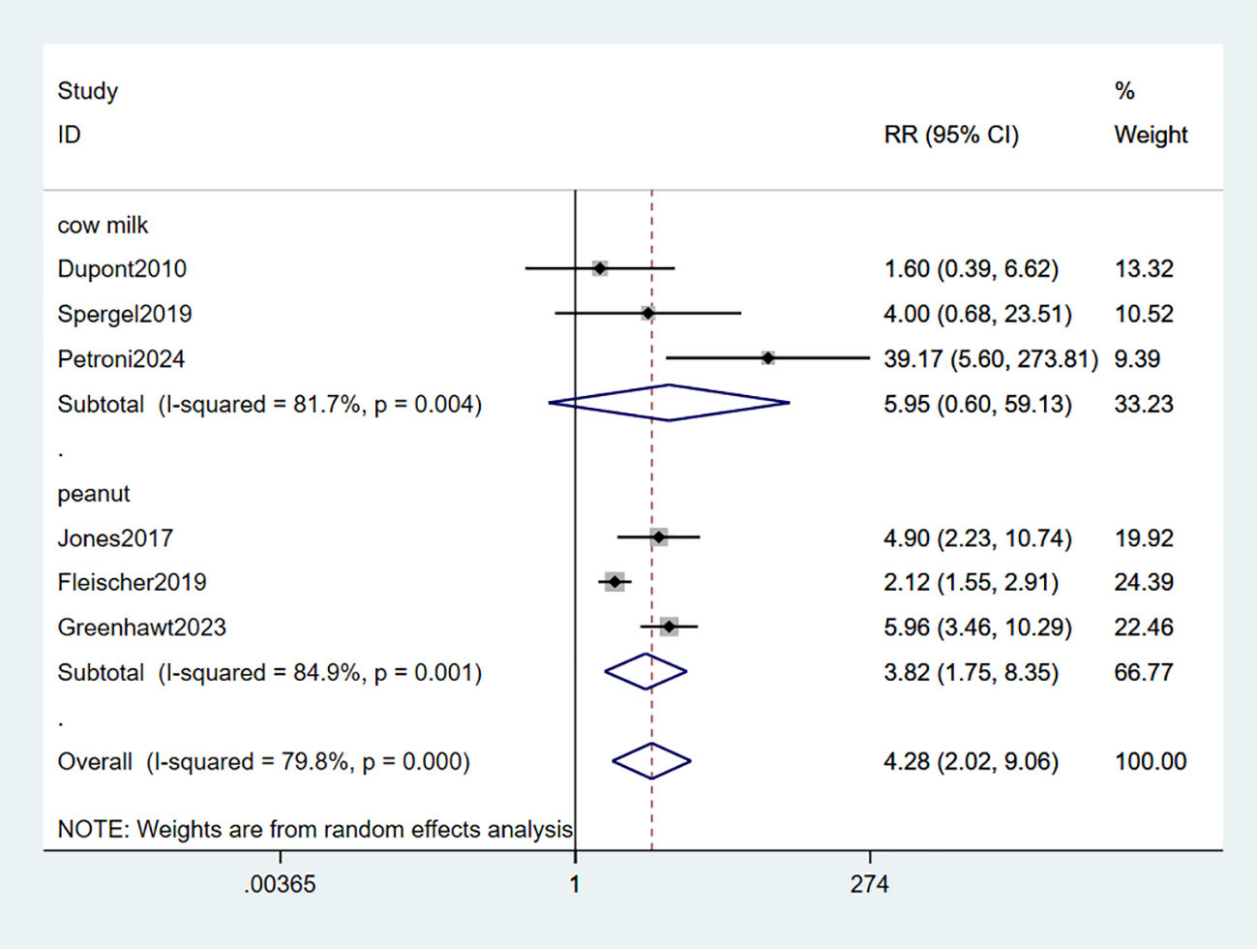
Forest plot of LARs.

Six studies observed adverse reactions in the local area (LARs) (33–37, 39). The combined data indicated that EPIT significantly increased the risk of local reactions (RR: 4.28, 95% CI: 2.02-9.06, P < 0.01, I2 = 79.8%) with substantial heterogeneity compared to placebo. Among these studies, three studies involving milk did not demonstrate statistical significance (RR: 5.95, 95% CI: 0.6-59.13, P < 0.01, I^2^ = 81.7%). However, the subgroup analysis focusing on peanuts revealed a significant association with LARs risk (RR: 3.83, 95% CI: 1.75-8.35, P < 0.01, I^2^ = 84.9%) (low certainty evidence; **Figure 5**).

#### 3.2.4 Systemic adverse reactions

**Figure 6:**
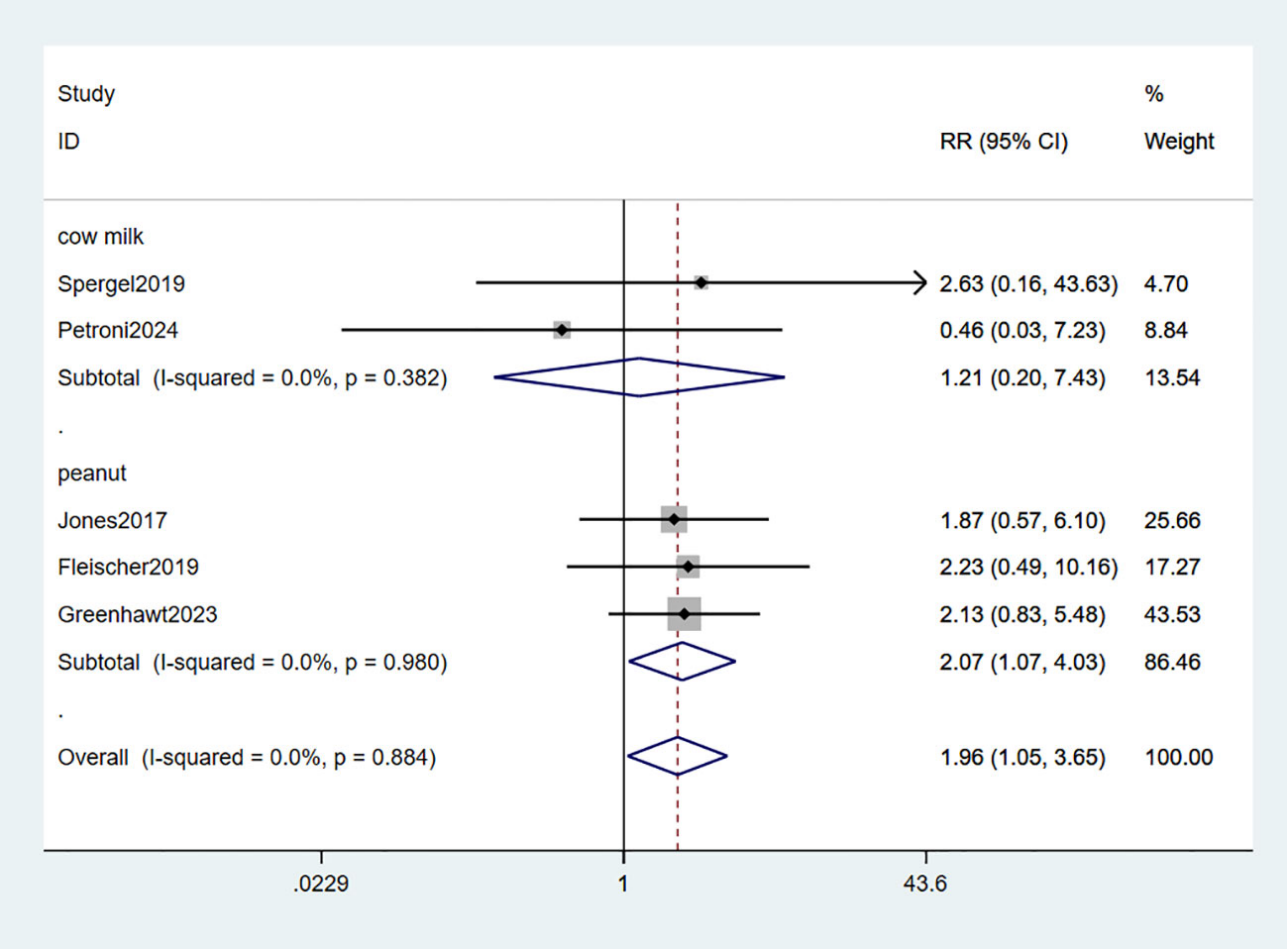
Forest plot of SARs.

Systemic adverse reactions (SARs) were reported in five studies (34–37, 39). The meta-analysis revealed a significant increase in the risk of systemic reactions with EPIT compared to placebo, exhibiting a relative risk of 1.96 and a 95% confidence interval ranging from 1.05 to 3.65 (P = 0.884, I^2^ = 0%). Notably, three studies focusing on peanuts demonstrated a statistically significant association with SARs risk (RR: 2.07, 95% CI: 1.07-4.03, P = 0.980, I^2^ = 0%), while two other milk-related studies did not reach statistical significance (RR: 1.21, 95% CI: 0.20-7.43, P = 0.382, I^2^ = 0%). Importantly, minimal observed heterogeneity ensures robust evidence (**Figure 6**).

#### 3.2.5 Serious adverse events

**Figure 7:**
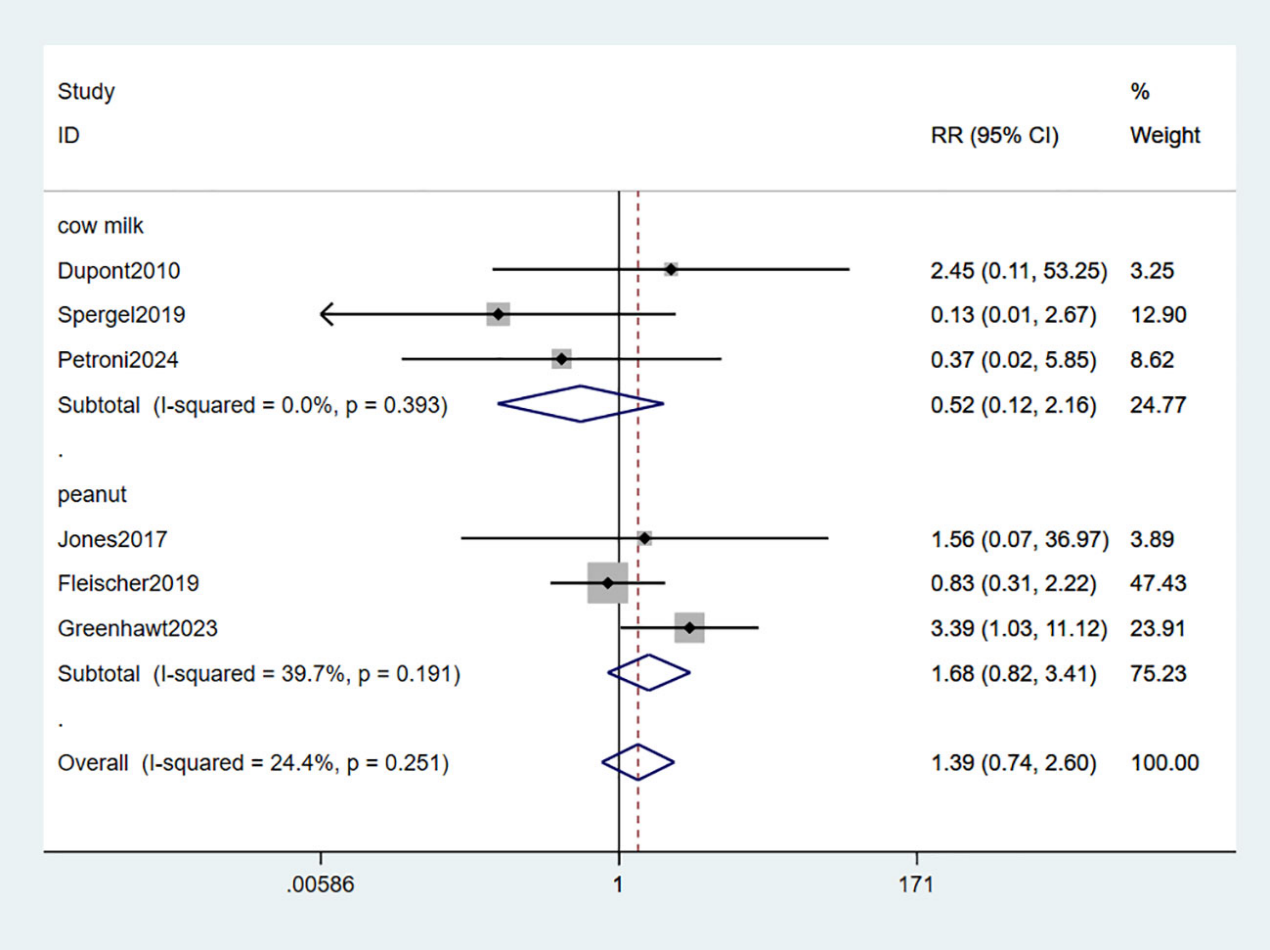
Forest plot of SAEs.

Six studies reported serious adverse events (SAEs) (33–37, 39). The meta-analysis findings revealed no statistically significant differences in the risk of SAEs between EPIT and placebo (RR: 1.39, 95% CI: 0.74-2.60, P = 0.251, I^2^ = 24.4%). Neither the peanut group (RR: 1.68, 95% CI: 0.82-3.41, P = 0.191, I^2^ = 39.7%) nor the milk group (RR: 0.52, 95% CI: 0.12-2.16, P = 0.393, I^2^ = 0%) exhibited any risk of experiencing SAEs (high certainty evidence; **Figure 7**).

### 3.3 Secondary outcome

#### 3.3.1 Epinephrine use

**Figure 8:**
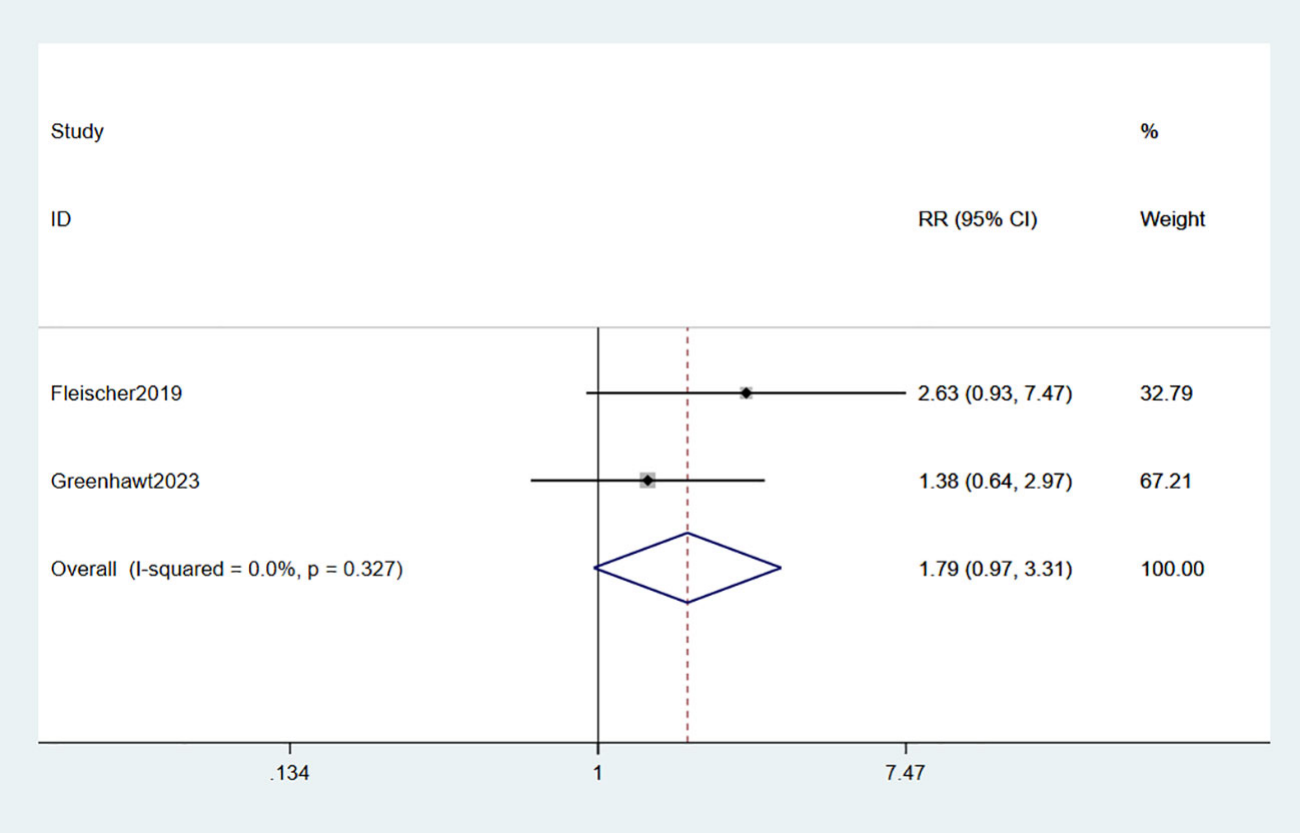
Forest plot of epinephrine use.

The administration of epinephrine was investigated in two studies involving a total of 718 participants (34, 35). Meta-analysis revealed that the use of epinephrine in EPIT did not show any significant evidence of increased risk compared to placebo (RR: 1.79, 95% CI: 0.97-3.31, p = 0.062, I^2^ = 0%) with minimal deviation from placebo (high certainty evidence; **Figure 8**).

#### 3.3.2 Allergic reaction of organ systems

The results of allergic reactions induced by EPIT in various organ systems are presented in **Table 2**. No conclusive evidence has been found to indicate a higher likelihood of adverse reactions occurring in the organ system when compared to the placebo group.

**Table 2:** Anaphylactic reactions stratified by organ systems.

Organ systems	*Sample size*	*RR (95% CI)*	*I ^2^, %*
Skin and subcutaneous tissue disorders	598 participants (4 RCTs)	1.16 (0.63–2.13)	67.1
Gastrointestinal disorders	598 participants (4 RCTs)	1.12 (0.86–1.44)	0
Eyes disorders	382 participants (2 RCTs)	0.91 (0.50–1.65)	0
Injury, poisoning and procedural complication	382 participants (2 RCTs)	0.91 (0.64–1.30)	0
Infections and infestation	580 participants (3 RCTs)	1.07 (0.96–1.20)	0
Respiratory, thoracic and mediastinal disorders	580 participants (3 RCTs)	1.02 (0.83–1.26)	0

RR, risk ratio; RCTs, randomized controlled trials.

### 3.4 Subgroup analysis

The study conducted subgroup analyses to investigate the impact of allergen type, therapeutic dosage, and treatment duration on the primary outcome. Additionally, desensitization was assessed for cow milk versus peanut allergens. Epidermal peanut immunotherapy demonstrated efficacy in both high-dose (> 100 µg) (RR: 2.77, 95% CI: 1.87-4.11, I² = 0%) and low-dose (≤ 100 µg) groups (RR: 2.91, 95% CI: 1.75-4.86, I² = 0%). However, no significant improvement in the efficacy of epidermal milk immunotherapy was observed in either the high-dose groups (> 300 µg) (RR: 1.50, 95% CI: 0.88-2.57, I² = 9.6%) or low-dose groups (≤ 300 µg) (RR: 1.42, 95% CI: 0.98-2.05, I² = 0%). Furthermore, subgroup analyses based on the durations (≥ 1 year versus < 1 year) indicated that only for durations ≥ 1 year did tolerance exhibit a statistically significant enhancement (RR: 2.12, 95% CI: 1.74-2.59, I² = 36%). Regarding LAR, subgroup analyses revealed that epidermal peanut immunotherapy alone had a significantly higher risk of LAR (RR: 3.82, 95% CI: 1.75-8.35, I² = 84.9%). The same trend was observed for SAR as well. Subgroup analyses were performed on the primary outcome and are presented in **Table 3**.

**Table 3:** Subgroup analyses.

	*Sample size*	*RR (95% CI)*	*I ^2^, %*
Desensitization			
Allergen types			
Cow milk	236 participants (3RCTs)	1.54 (0.99–2.40)	0
Peanut	905 participants (4RCTs)	2.29 (1.83–2.86)	0
Dose			
Peanut≤100ug	133 participants (2RCTs)	2.91 (1.75–4.86)	31.92
Peanut>100ug	437 participants (3RCTs)	2.77 (1.87–4.11)	68.08
Milk≤300ug	98 participants (2RCTs)	1.42 (0.98–2.05)	65.33
Milk>300ug	80 participants (3RCTs)	1.50 (0.88–2.57)	34.67
Treat duration			
<1years	38 participants (2RCTs)	5.09 (0.74–35.09)	0
≥1years	1103 participants (5RCTs)	2.09 (1.71–2.55)	36
LAR			
Allergen types			
Cow milk	236 participants (3RCTs)	5.95 (0.60–59.13)	81.7
Peanut	905 participants (4RCTs)	3.82 (1.75–8.35)	84.9
SAR			
Allergen types			
Cow milk	236 participants (3RCTs)	2.07 (1.07–4.03)	0
Peanut	905 participants (4RCTs)	1.21 (0.20–7.43)	0
ES			
Allergen types			
Cow milk	216 participants (2RCTs)	0.55 (0.09–3.55)	0
Peanut	718 participants 2RCTs)	1.73 (0.84–3.53)	0

RR, risk ratio; RCTs, randomized controlled trials; LAR, Local adverse reactions; SAR, Systemic adverse reactions; ES, epinephrine use.

### 3.5 Sensitivity analysis and publication bias

Sensitivity analyses were conducted on the primary findings by excluding studies with significant or uncertain potential for bias in terms of selection, performance, detection, or attrition. The exclusion of all studies with a high or unclear risk of bias did not impact the outcome measures (**Supplementary Material**).

The results of Egger’s test did not reveal any statistically significant indication of publication bias in relation to desensitization (p = 0.957) or the primary outcomes TRAE (p = 0.643), LAR (p = 0.861), SAR (p = 0.425), and SAE (p = 0.073). Therefore, there is no substantial evidence suggesting the presence of significant publication bias concerning these outcomes.

## 4 Discussion

Our analysis suggests that EPIT holds promise for improving tolerance in pediatric patients with food allergies. Subgroup analysis indicates a potential benefit of EPIT in enhancing peanut allergy tolerance among children, while its impact on cow’s milk allergy remains inconclusive. Furthermore, our findings suggest a possible association between EPIT and adverse events such as TRAE, LAR, SAR, etc. However, no statistically significant increase was observed in the risk of SAEs or epinephrine usage associated with EPIT.

The effectiveness of EPIT in the treatment of food allergy observed in our study aligns with previous meta-analyses conducted on this topic. Xiong et al. (28) performed a meta-analysis that included ten RCTs investigating the efficacy of EPIT for food allergy, and their findings indicated significant benefits in terms of desensitization specifically for peanut allergy. Additionally, subgroup analyses based on different age groups confirmed notable improvements in tolerance among children aged below 12 years. The authors also noted an increased likelihood of local skin reactions associated with EPIT but did not identify any significant correlations with TRAE, SAR, SAEs, or the use of rescue medications. Banatwala et al. (29) conducted a meta-analysis to evaluate the efficacy and safety of EPIT in individuals with peanut allergies. Their results revealed that only the higher dosage group (250 µg) exhibited significantly greater desensitization compared to placebo, while no significant difference was observed in the lower dosage group (100 µg). However, they noted a notable increase in both local and systemic adverse events associated with EPIT treatment. Furthermore, individuals undergoing EPIT were more likely to require rescue medications such as epinephrine and topical corticosteroids. On the other hand, Alvarez-Florian et al. (40) performed a systematic review focusing on children and adolescents with milk allergy but found insufficient evidence to determine the efficacy of EPIT for cow’s milk allergy.

The urgent requirement for safe and effective treatments is underscored by the detrimental impact of food allergy on patients’ physical, psychological, and social well-being. Our study found that EPIT treatment was associated with the incidence of TRAEs, LARs and SARs. However, it did not lead to an increased occurrence of SAEs or necessitate additional epinephrine use. Current clinical research focuses on food allergen-specific immunotherapy through OIT, SLIT, or EPIT routes. Compared to SLIT and EPIT, OIT necessitates a higher maintenance dose of food protein and is associated with an elevated incidence of systemic adverse events. While SLIT exhibits safety, its efficacy is constrained by the sublingual administration of low-dose allergen. In contrast, EPIT surpasses both SLIT and EPIT in terms of safety and tolerability while demonstrating moderate therapeutic effects (41). Consequently, EPIT demonstrates a favorable safety profile. In the context of food allergy immunotherapy, when considering the augmentation of immune tolerance, both the potential risks and benefits should be taken into account alongside patient expectations. Patients who possess high expectations for achieving enhanced immunization thresholds and are willing to accept the associated risk or cost of side effects may be suitable candidates for OIT. Conversely, patients with heightened safety expectations might find EPIT more appropriate (42).

The phenomenon of desensitization was primarily observed in younger children, which is consistent with the superior outcomes of OIT observed in this age group (16, 43). This observation can be attributed to the enhanced permeability of water-soluble allergens through the skin. In the Viaskin epidermal delivery system, water-soluble allergens are absorbed into both the epidermis and dermis layers (44), where they encounter dendritic cells. Subsequently, these cells migrate to nearby lymph nodes, thereby triggering immune responses (45, 46). Children have different skin characteristics compared to adults, including differences in structure, barrier function, and composition (47, 48). Furthermore, the outermost layer of children retains higher levels of moisture and exhibits a faster rate of water movement compared to that of adults (48, 49). Due to the presence of only about 15 layers in children’s outermost layer versus 25 layers in adults’, it can be inferred that water-soluble allergens are more likely to penetrate through less developed outermost layers in children when compared to adults. Additionally, the age disparity in immune mechanisms also serves as a significant factor contributing to the heightened desensitization observed in younger children. The correlation between the gradual postnatal increase of IgE levels and the persistence of food allergy in infants (50, 51) further underscores this relationship. Notably, a substantial expansion of IgE epitope recognition occurs after reaching two years of age (52, 53). Consequently, the immune response to food allergens remains immature during the early childhood. Moreover, the functional abilities of IgE-producing B cells are also immature in the first few years of life. Notably, age-dependent developmental changes in peripheral blood B cell pool composition exhibit their most pronounced effects within the initial five years of life, while the number of naive B cells gradually decreases to adult levels by 10-15 years of age (54). Therefore, altering the balance of food allergen tolerance becomes more challenging following the maturation of B-cell function, and this initial immaturity of the immune response to food allergens may constitute another significant factor contributing to the diminished efficacy of EPIT in older children and adults.

This review offers several advantages. Firstly, it represents the initial meta-analysis that evaluates the effects and safety of EPIT for food allergy in children, providing robust evidence to support EPIT as an innovative allergen immunotherapy. Secondly, all included studies are RCTs, enhancing the credibility of our findings due to their high quality. Thirdly, we conducted subgroup analyses based on various study characteristics to investigate potential sources of significant heterogeneity observed in local adverse effects. There are several noteworthy constraints in our study. Firstly, the included studies had a limited number and sample size, potentially introducing random errors. Secondly, the EPIT patches utilized in this study were exclusively sourced from Viaskin (DBV Technologies), which also influenced the trial design, potentially limiting the generalizability of the findings to other EPIT products developed by different manufacturers. Additionally, there is a lack of studies investigating EPIT for food allergies beyond peanut and milk. Thirdly, due to variations in EPIT time and dosage across different studies, conducting subgroup analysis to determine the most suitable intervention time and dose posed challenges. Another limitation is that certain studies only encompassed a subset of young adults.

In conclusion, this systematic review and meta-analysis provide compelling evidence suggesting that EPIT may potentially induce desensitization of peanut allergy in children. The efficacy of EPIT for cow milk allergy remains uncertain, and current evidence does not support the utilization of EPIT treatment with milk patches in pediatric clinical practice; however, it is noteworthy that EPIT also exhibits mild to moderate side effects. Consequently, the safety profile of EPIT is considered favorable. Given the limited number of studies and variations in research methodologies, these findings should be cautiously interpreted. Further well-designed RCTs with larger sample sizes are warranted to investigate the benefits and adverse events associated with EPIT in pediatric populations as well as other allergic conditions.

## Data Availability

The original contributions presented in the study are included in the article/**Supplementary Material**. Further inquiries can be directed to the corresponding authors.

## Supplementary material

Supplementary Figure 1 Sensitivity analysis of desensitization.

Supplementary Figure 2 Sensitivity analysis of TRAEs.

Supplementary Figure 3 Sensitivity analysis of LARs.

Supplementary Figure 4 Sensitivity analysis of SARs.

Supplementary Figure 5 Sensitivity analysis of SAEs.
