# Supplementary figures and images for: Epithelial immunotherapy for food allergy in children: a systematic review and meta-analysis
Source: Front Immunol. 2024 Dec 23;15:1510653. doi: 10.3389/fimmu.2024.1510653 (PMC11700978; doi:10.3389/fimmu.2024.1510653)

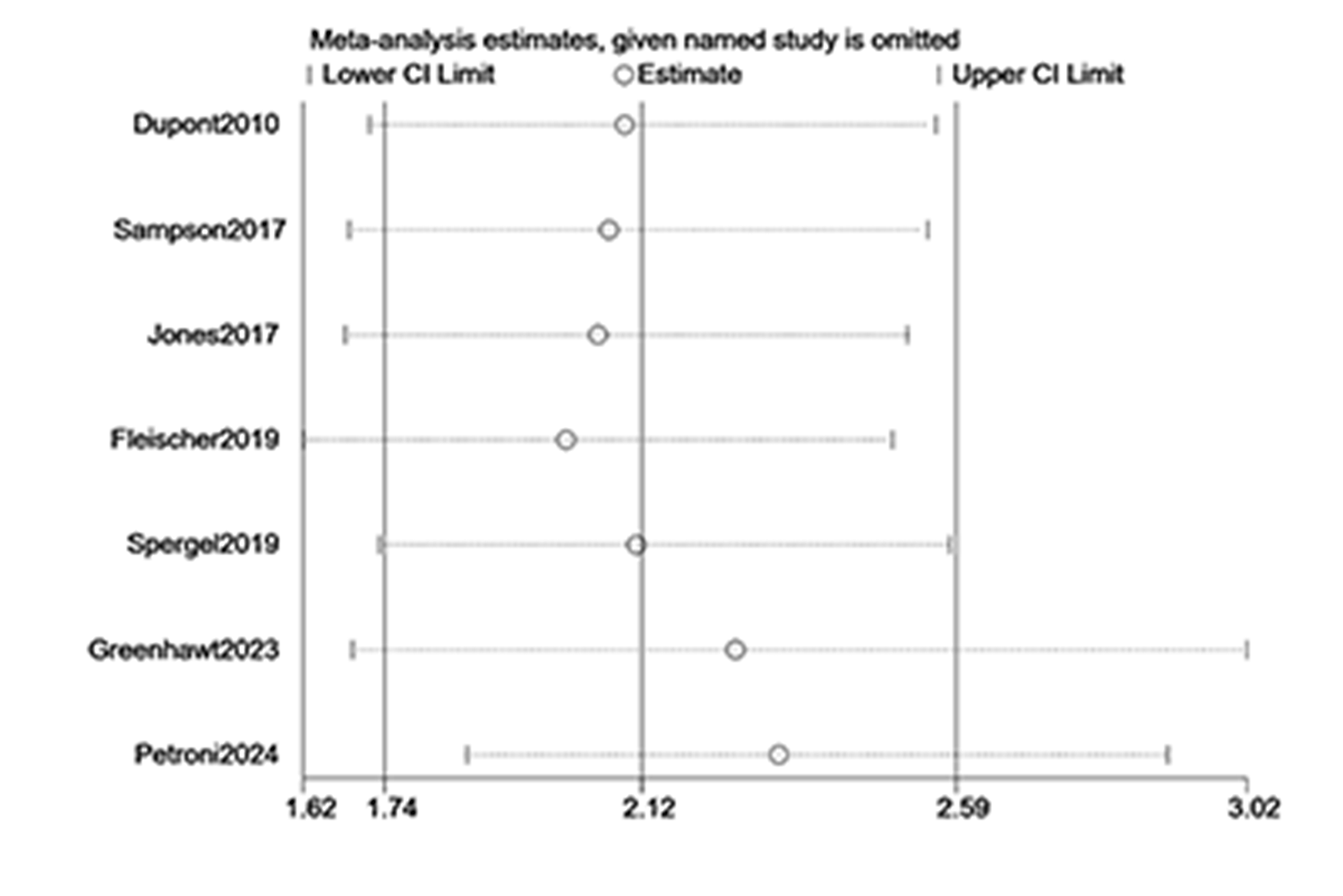

Supplement: Supplementary Figure 1 — Sensitivity analysis of desensitization. [file Image1.tif]

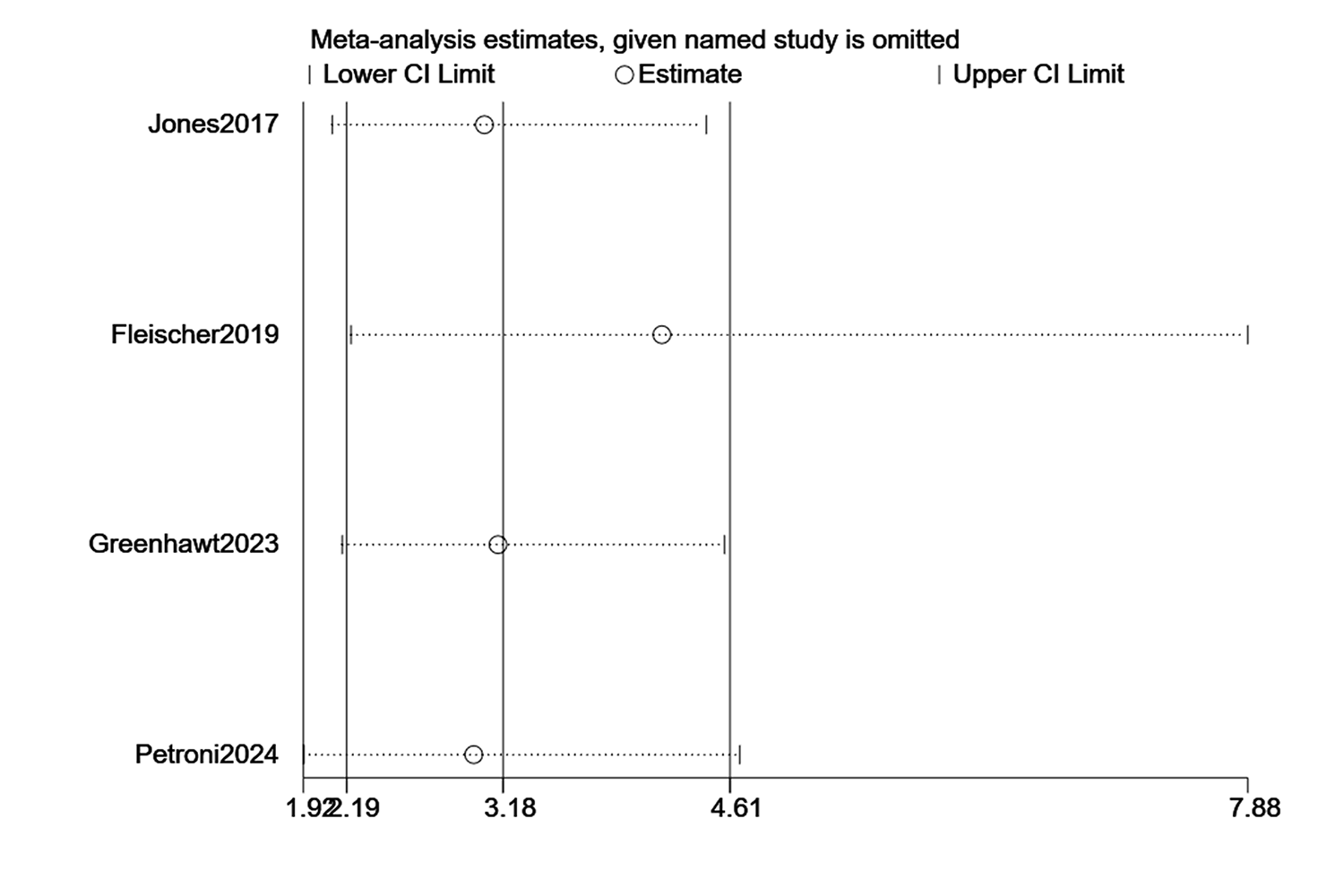

Supplement: Supplementary Figure 2 — Sensitivity analysis of TRAEs. [file Image2.tif]

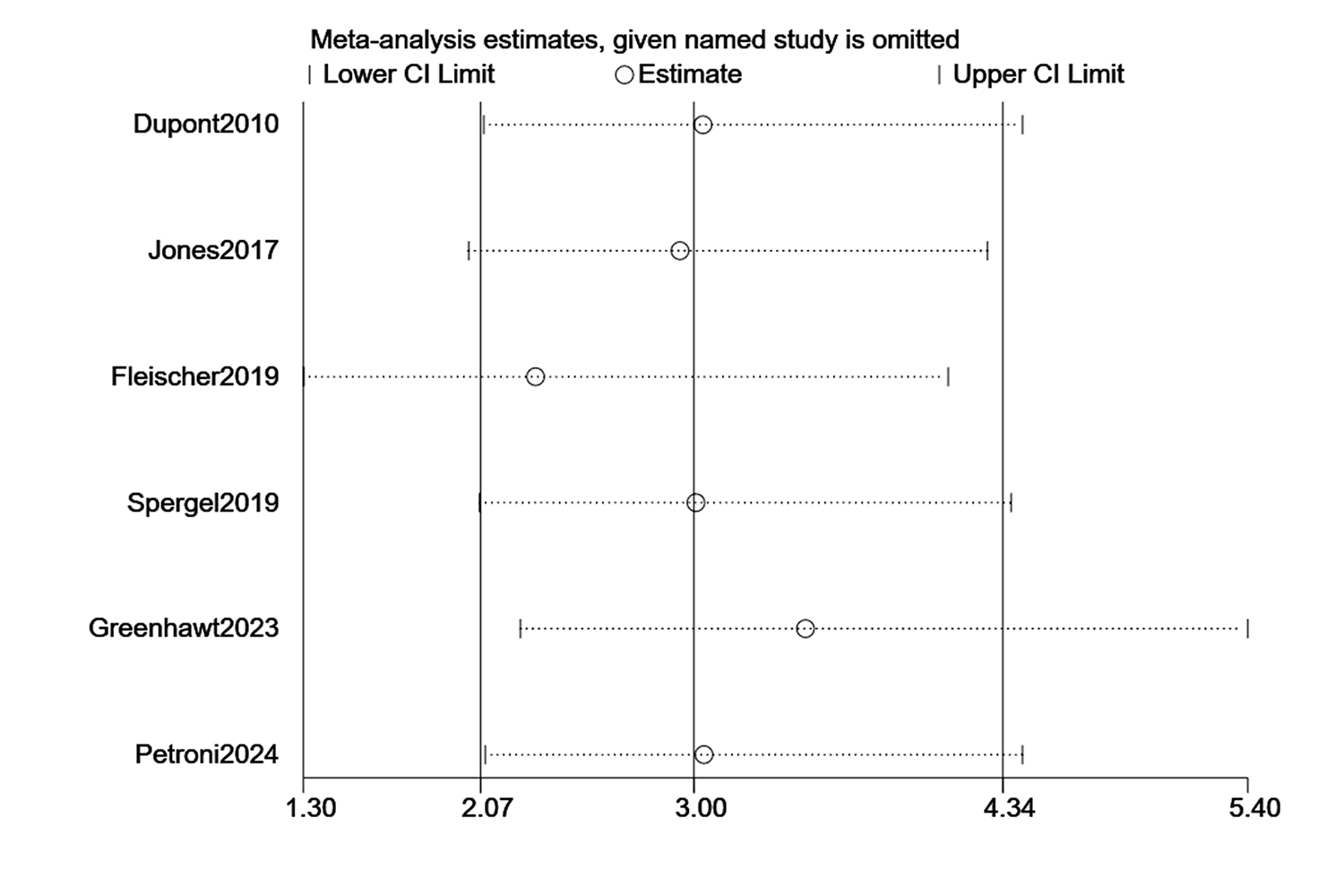

Supplement: Supplementary Figure 3 — Sensitivity analysis of LARs. [file Image3.tif]

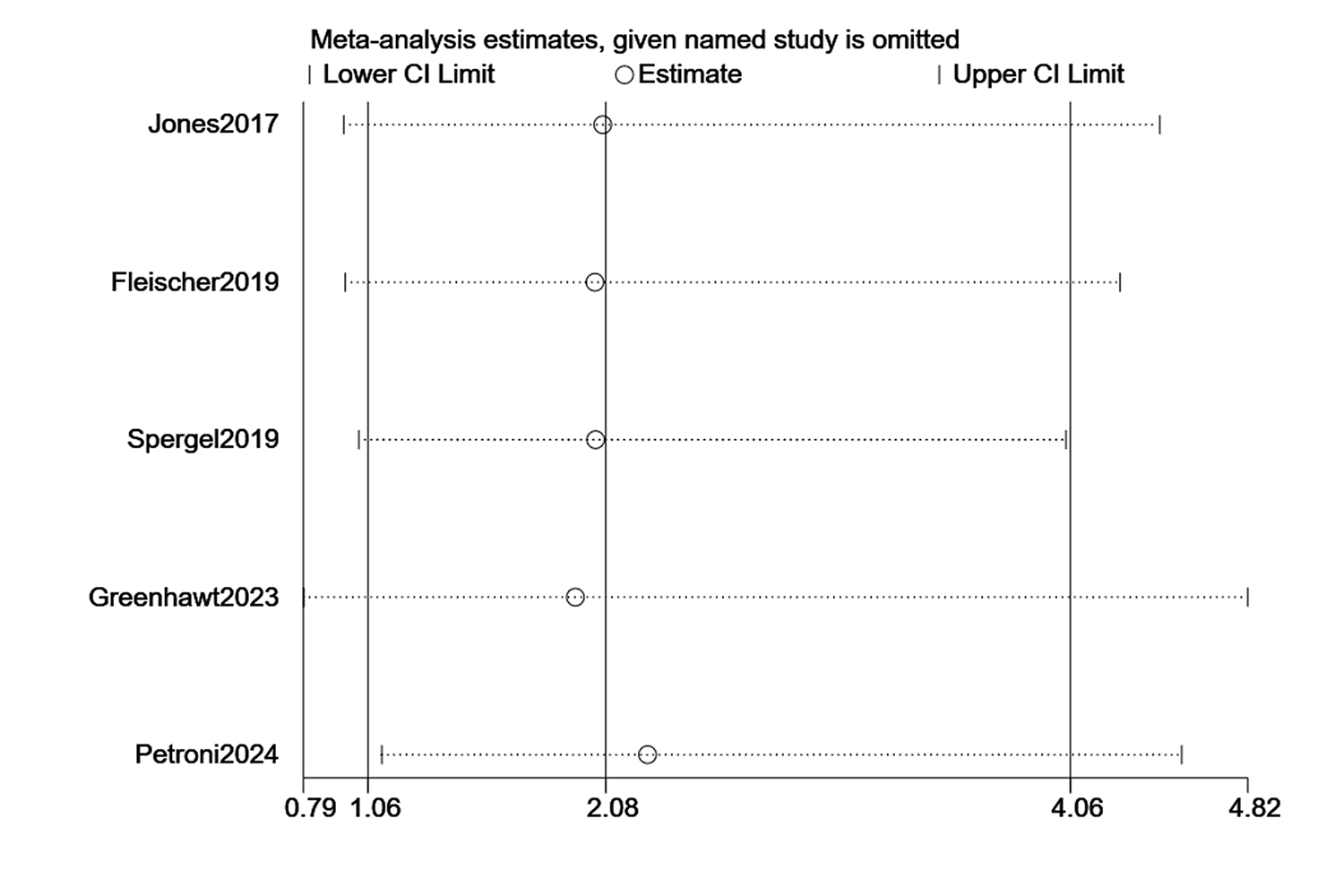

Supplement: Supplementary Figure 4 — Sensitivity analysis of SARs. [file Image4.tif]

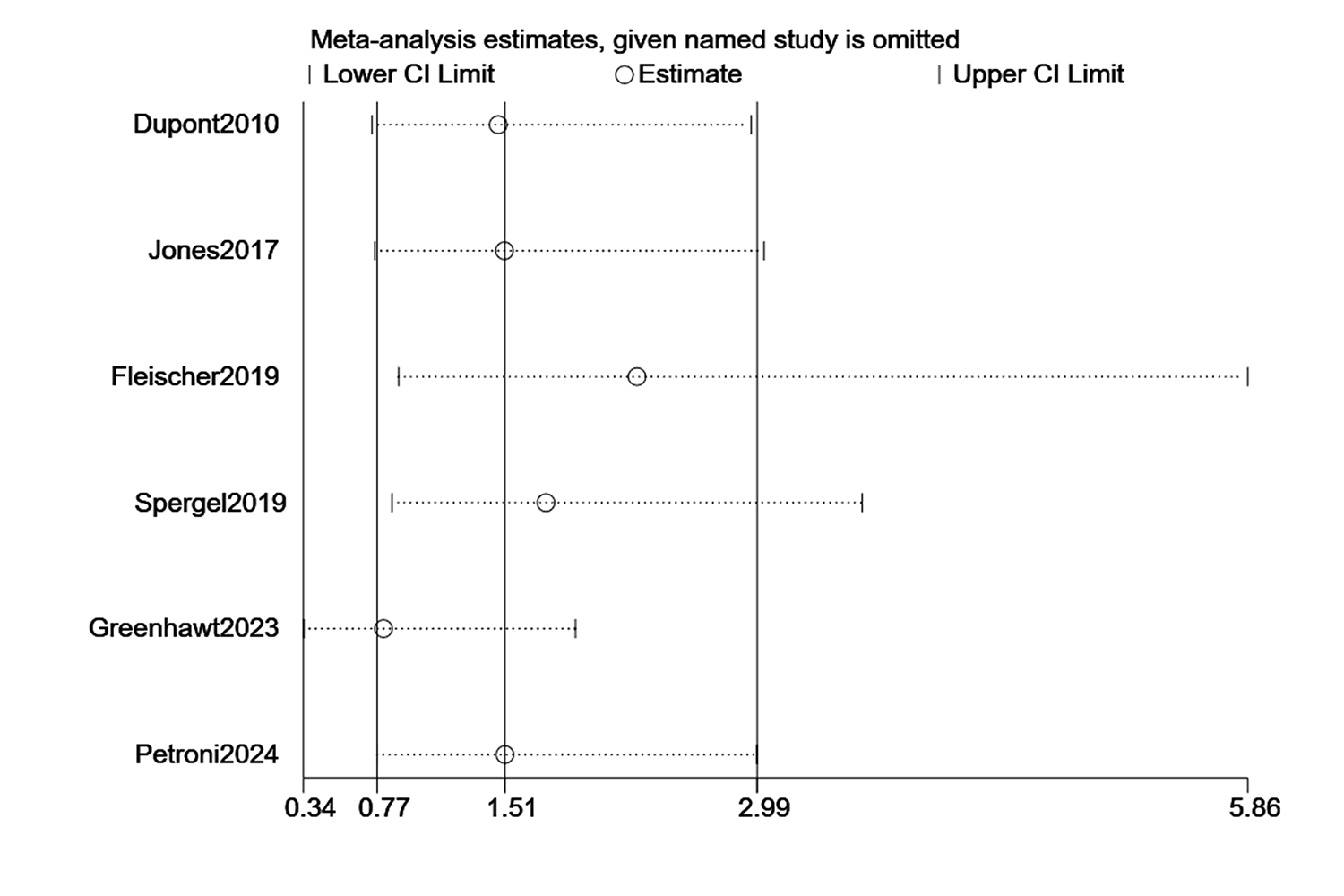

Supplement: Supplementary Figure 5 — Sensitivity analysis of SAEs. [file Image5.tif]
